# Superior vena cava coil left alone in the innominate vein: successful removal by a combined approach via jugular and femoral veins

**DOI:** 10.1093/ehjcr/ytae077

**Published:** 2024-02-05

**Authors:** Kenji Kuroki, Yukio Sekiguchi, Kazutaka Aonuma, Akira Sato

**Affiliations:** Department of Cardiology, University of Yamanashi, 1110 Shimokato, Chuo, Yamanashi 409-3898, Japan; Department of Cardiology, Sakakibara Heart Institute, Fuchu, Tokyo, Japan; Department of Cardiology, Saiseikai Mito Hospital, Mito, Ibaraki, Japan; Department of Cardiology, University of Yamanashi, 1110 Shimokato, Chuo, Yamanashi 409-3898, Japan

**Figure:**
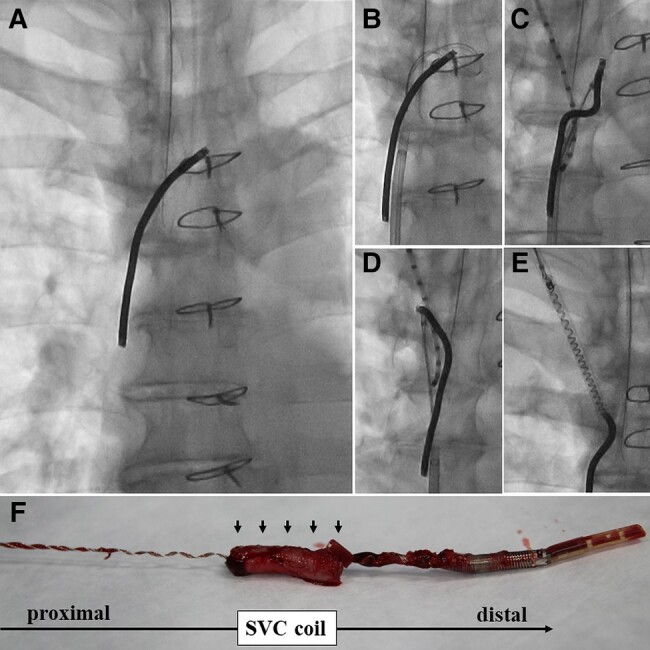


The extraction of migrating leads is technically challenging, especially when a superior vena cava (SVC) coil was left alone in the innominate vein.

A 56-year-old man with Brugada syndrome developed a delayed cardiac tamponade due to perforation of the right ventricle (RV) defibrillation lead (Riata 1580, Abbott) implanted 10 years ago. The RV was surgically repaired, and the RV lead was resected at the right atrium (RA) level. One year later, he was referred to our hospital to undergo lead extraction due to pocket infection. A locking stylet was developed at the lead’s distal end, which could not be captured by a snare catheter inserted via the femoral access because the distal end was resected just below the level adherent to the RA. A 14-Fr laser sheath was advanced to one-third of the SVC coil when the lead was broken (*Panel A*). The snare catheter was advanced to the innominate vein to grab the proximal end of the SVC coil (*Panel B*). The proximal coil was seized using the snare with the support of the deflectable catheter inserted from the internal jugular vein (*Panel C*), and the coil was moved into the jugular vein (*Panel D*; Supplementary material online, *Video S1*). The coil was grabbed with the snare catheter advanced via the internal jugular access, but the coil was easily untied gradually by pulling it (*Panel E*; Supplementary material online, *Video S2*). With continuous mild traction, the entire coil was finally pulled out of the body. The SVC coil with massive adhesion (arrows) had been untied almost entirely (*Panel F*).

## Supplementary Material

ytae077_Supplementary_Data

## Data Availability

The data sets used and/or analysed during the current study are available from the corresponding author upon reasonable request.

## Supplementary material

**Consent**: The authors confirm that witnessed verbal consent for submission and publication of this case report, including images and associated text, has been obtained from the patient. This has been discussed with the editors.

**Funding**: None declared.

